# Impaired immune function accompanies social evolution in spiders

**DOI:** 10.1098/rsbl.2022.0331

**Published:** 2022-12-21

**Authors:** Jesper Bechsgaard, Tove Hedegaard Jorgensen, Anne Katrine Jønsson, Mads Schou, Trine Bilde

**Affiliations:** ^1^ Department of Biology, Aarhus University, Aarhus, Denmark; ^2^ Department of Biology, Lund University, Lund, Sweden

**Keywords:** immunity, sociality, comparative genomics, social spider, subsocial spider, haemolymph assay

## Abstract

An efficient immune system is essential to the survival of many animals. Sociality increases risk of pathogen transmission, which should select for enhanced immune function. However, two hypotheses instead predict a weakened immune function: relaxed selection caused by social immunity/protection, and reduced efficacy of selection due to inbreeding, reproductive skew and female bias in social species that reduce effective population size and accelerate genetic drift. We assessed the effect of social evolution on immune function in a comparative study of two social spider species and their closely related subsocial sister species (genus *Stegodyphus*). The haemolymph of social species was less efficient in inhibiting bacterial growth of the potentially pathogenic bacteria *Bacillus subtilis* than that of subsocial species. Reduced efficacy of selection in social species was supported by comparative genomic analysis showing substantially elevated non-synonymous substitutions in immune genes in one of the social species. We propose that impaired immune function results from reduced efficacy of selection because the evolution of sociality in spiders is accompanied by demographic processes that elevate genetic drift. Positive feedback between pathogen-induced local extinctions and the resulting elevation of genetic drift may further weaken responses to selection by pathogens, and threaten species persistence.

## 1. Introduction

The physiological responses of immune systems are critical for reducing detrimental effects of pathogen infections. In most animals, the genes that control a physiological immune response are either highly conserved to maintain essential functions in signalling pathways, or under positive and/or balancing selection to recognize and eliminate a diversity of invading pathogens e.g. [1,2]. Group living animals experience increased risk of pathogen transmission due to frequent and close contact between individuals e.g. [3], suggesting that selection for efficient immune responses is stronger in social than in non-social species. Two conditions of social lifestyle may however interfere with the adaptive evolution of immune genes and obscure predictions on their substitution patterns and the resulting immune response efficiency. Firstly, many social species have evolved social protection from infection, including behavioural responses, which collectively reduce disease transfer [4]. Social protection includes the collective increase in body temperature in honeybees [5], the use of antimicrobial tree resins in ant nests [6] or the association with microbial symbionts in spiders [7] and possibly aphids [8]. Effective social protection should relax selection on physiological immune responses [9–12]; in line with observations of relaxed selection on behavioural responses owing to group living [13–16]. Secondly, genetic drift may be particularly strong in some social species due to low effective population size (Ne) [17–20]. This causes random loss of genetic diversity by drift as well as reduced efficacy of selection [21,22], which may impair immune gene function.

In general, the arthropod immune system is innate and therefore relies on immune gene variants to recognize and eliminate invading pathogens. The first line of defence is constitutive and includes the actions of haemocytes and rapidly activated enzyme cascades that may effectively clear infection [23,24]. The second line of defence consists of induced responses (signal transduction) that occur when blood cell membrane receptor proteins bind to certain surface proteins of the pathogen and initiate signalling pathways like Toll and Imd, resulting in the production of antimicrobial peptides [1,23]. There is however substantial variation in immune system responses across taxonomic groups. In spiders, several genes that encode proteins involved in the signal transduction pathways seem to be missing [25], supporting the presence of constitutively expressed antimicrobial peptides [26,27].

**Figure 1.**
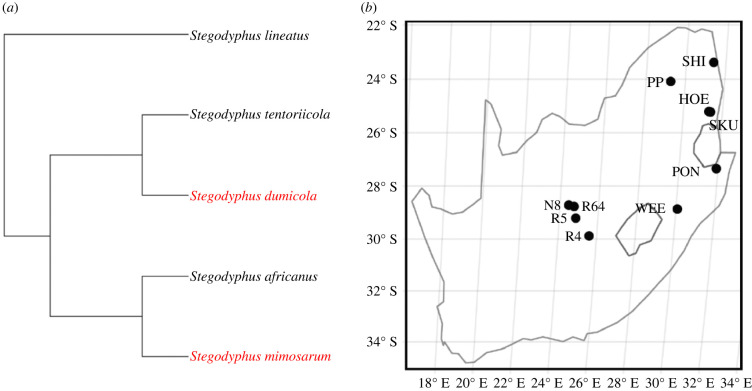
Phylogenetic (*a*) and geographical (*b*) information on sampled species and populations. *Stegodyphus lineatus* is included as outgroup in (*a*). *S. tentoriicola* was sampled at R4, R5, N8 and R64, *S. dumicola* at PON, SKU, WEE, *S. africanus* at HOE, PON, SHI, SKU and *S. mimosarum* at PON, PP, SKU, WEE. Social species are marked in red and subsocial species in black.

Here we test the effect of sociality on immune function assayed by growth inhibition of two bacteria in two independently evolved social spiders and their respective subsocial sister species of the genus *Stegodyphus* [28] (figure 1*a*). Social *Stegodyphus* spiders form permanent groups in communal nests, with obligate inbreeding, female-biased sex ratio (approx. 1 : 8), and reproductive skew [19], factors that all contribute to reduce effective population size. Low population genetic diversity and elevated rates of genome-wide non-synonymous substitutions compared to their subsocial sister species indicate strong effect of drift and reduced efficacy of selection in social lineages [22,29,30]. We first investigated if haemolymph of *Stegodyphus* spiders inhibits the growth of two surrogate pathogens, the Gram-negative *Escherichia coli* and the Gram-positive *Bacillus subtilis*. Then we investigated whether social species show a relatively *stronger* immune response (stronger inhibition) than subsocial species as expected if sociality and elevated risk of transmission selects for a more efficient immune response; or alternatively, whether social species show relatively *weaker* inhibition, as predicted under lower efficacy of selection and/or relaxed selection due to social protection. We additionally investigated whether selection at the molecular level differs between social and subsocial species by comparing substitution patterns (dN/dS) in genes involved in the immune signal transduction pathways.

## 2. Material and methods

Multiple individuals were sampled from 3–4 populations of each of the social species *S. mimosarum* and *S. dumicola* and subsocial *S. africanus* and *S. tentoriicola* in January 2015 in South Africa (figure 1). Individuals were kept at 27°C and a 13 L : 11 D light/dark cycle until use in *in vitro* immune assays in mid-April 2015. Two experiments were conducted, with *Bacillus subtilis* DSM10 and *Escherichia coli* OP50 respectively, both in NB medium (Scharlau, Spain). Haemolymph was extracted from fully hydrated, sterilized and anaesthetized female spiders by piercing a book lung and collecting 2 µl from the emerging droplet. Each 2 µl haemolymph sample was randomly distributed to wells containing bacterial medium (100 µl each) in 96-well plates. On average 6.4 ± 3.8 (s.d.) haemolymph samples were included per run to keep the time from adding haemolymph to incubation short. Plates were incubated at 27°C except during measurements at approximately 20°C. Each plate contained two treatment controls; one sample of NB medium without haemolymph and one with Ringer's solution, the latter to mimic the haemolymph salt composition. Sample sizes for assays were: *B. subtilis*: *n_africanus_* = 37, *n_mimosarum_* = 51, *n_tentoriicola_* = 14, *n_dumicola_* = 28, *n*_NB_ = 27, *n*_NB+Ringer_ = 27 and *E. coli*: *n_africanus_* = 35, *n_mimosarum_* = 52, *n_tentoriicola_* = 14, *n_dumicola_* = 24, *n*_NB_ = 24, *n*_NB+Ringer_ = 24. Before the addition of haemolymph, bacterial concentration in each well was measured as light absorbance using an Epoch microplate spectrophotometer to standardize start concentrations. After addition of haemolymph the light absorbance was measured every 2nd minute from 0 to 16 min, every 5th minute from 20 to 50 min, and then at 60, 70, 80, 100 and 120 min.

In the analyses, we first examined the difference in bacteria growth of NB medium with and without Ringer's solution. We modelled absorbance values (Gaussian response variable) using the R-package lmerTest v. 3.1-3 [31] in R v. 4.0.5 [32], with the fixed effects Time (continuous, scaled but not centred), Medium (factorial, Ringer's or without Ringer's solution) and Bacteria (factorial, *E. coli* or *B. subtilis*) with full sets of interactions. We included plate identity as a random effect, with both random intercept across plates, and random slopes of each plate over time. We used Ringer as a control to test for inhibition of growth of each of the two bacteria caused by the haemolymph of four spider species since we found no effect on absorbance (electronic supplementary material, table S1). We constructed one model per spider species, with the fixed effects Time and Medium (factorial, Ringer or spider haemolymph), and with random intercept and slopes of plate identity over time. We checked whether our results were sensitive to potential nonlinearity by repeating all analyses of *B. subtilis* in two separate linear phases (0–16 min and 20–120 min, electronic supplementary material, table S1 and S2) *post hoc*.

To investigate if the haemolymph of social spiders differed from subsocial spiders in the inhibition of bacteria growth, we constructed models for each spider sister pair. These models contained the fixed effects Time and Social (Yes or No), and their interaction. As the structure of repeated sampling over time was similar across the two media, we could model random slopes and intercept of each haemolymph sample within a plate. We also added plate identity and population of origin as random intercepts. The statistical significance of model terms was estimated using *F*-tests by the Satterthwaite's approximation in lmerTest (type II ANOVA).

To investigate if selection at the molecular level differs between social and subsocial species, we used 23 immune genes annotated in the *S. mimosarum* genome [25]. Alignments consisting of orthologue sequences from *S. mimosarum*, *S. africanus* and *S. lineatus* were obtained from Bechsgaard *et al*. [29]. *Stegodyphus dumicola* sequences were extracted from the genome assembly published by Liu *et al*. [33] and *S. tentoriicola* sequences were extracted from an unpublished genome assembly. We used PAML 4.6 [34] to estimate dN/dS ratios averaged across sites for each branch separately, first for individual loci, and secondly for all loci concatenated. Ninety-five per cent confidence intervals were estimated from 1000 bootstrapped datasets using codeml. We analysed whether selection was relaxed or intensified in species with elevated dN/dS using RELAX [35].

## 3. Results

**Figure 2.**
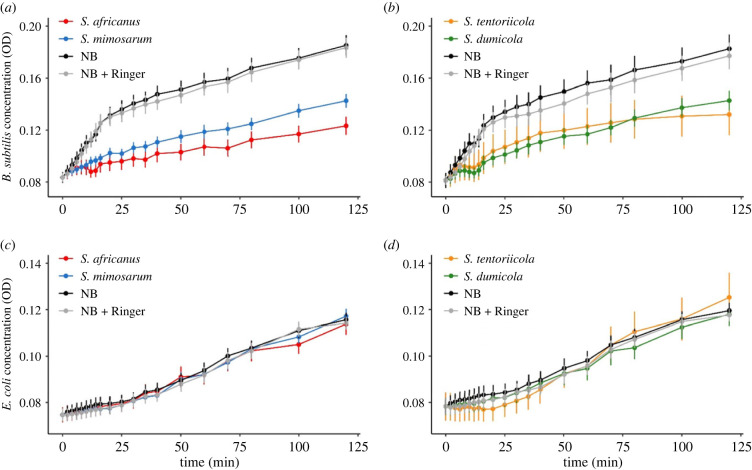
Growth of *Bacillus subtilis* (top panels) and *Escherichia coli* (lower panels) measured as changes to cell concentration in NB media with haemolymph from subsocial *Stegodyphus africanus* and its social sister species *S. mimosarum* (*a*,*c*) and from subsocial *S. tentoriicola* and its social sister species *S. dumicola* (*b*,*d*). Changes to cell concentration was estimated as mean absorbance (OD) (± S.E.)*.* Baseline measures of uninhibited bacterial growth were obtained in pure NB media (NB) and NB with Ringer's solution (NB + Ringer). Cell concentrations are standardized to Ringer's medium at time zero to illustrate differences in growth rates.

The haemolymph of all four spider species inhibited the growth of *B. subtilis* (electronic supplementary material, table S2), while none of the species inhibited the growth of *E. coli* (electronic supplementary material, table S3). The subsocial *S. africanus* showed stronger inhibition of *B. subtilis* growth than the social *S. mimosarum* (table 1, figure 2*a*). The result for the *S. tentoriicola*–*S. dumicola* comparison was inconclusive (table 1, figure 2*b*), although, the data could indicate stronger inhibition of *B. subtilis* growth by subsocial *S. tentoriicola* than by social *S. dumicola* after the first approximately 20 min. No differences in growth inhibition of *E.* coli among spider species were detected (table 2).

**Table 1.** Comparison of growth rates of *B. subtilis* in haemolymph from two sister-species pairs: subsocial *Stegodyphus tentoriicola*/social *S. dumicola* and subsocial *S. africanus*/social *S. mimosarum*.

pair	term	estimate (se)	d.f.	*F*	*p*
*S. tentoriicola* versus *S. dumicola*	intercept	0.098 (0.007)	—	—	—
	social (no)	0.006 (0.007)	1.31	0.9	0.337
	time_z	0.026 (0.003)	1.42	82.6	**<0**.**001**
	social (no):time_z	−0.004 (0.006)	1.42	0.6	0.458
*S. africanus* versus *S. mimosarum*	intercept	0.099 (0.006)	—	—	—
	social (no)	0 (0.005)	1.75	0	0.923
	time_z	0.023 (0.002)	1.88	143.9	**<0**.**001**
	social (no):time_z	−0.008 (0.003)	1.88	6.2	**0**.**015**

**Table 2.** Comparison of growth rates of *E. coli* in haemolymph from two sister-species pairs: subsocial *Stegodyphus tentoriicola*/social *S. dumicola* and subsocial *S. africanus*/social *S. mimosarum*.

pair	term	estimate (s.e.)	d.f.	f	*p*
*S. tentoriicola* versus *S. dumicola*	intercept	0.079 (0.006)	—	—	—
	social (no)	0.008 (0.006)	1.10	2.3	0.16
	time_z	0.017 (0.002)	1.38	182.7	**<0**.**001**
	social (no):time_z	0.004 (0.003)	1.38	1.8	0.193
*S. africanus* versus *S. mimosarum*	intercept	0.08 (0.004)	—	—	—
	social (no)	0.001 (0.003)	1.23	0.2	0.657
	time_z	0.017 (0.001)	1.87	508.2	**<0**.**001**
	social (no):time_z	−0.001 (0.002)	1.87	0.6	0.439

Estimated dN/dS ratio averaged across sites were significantly higher in the social *S. mimosarum* (0.1876 (95% CI: 0.1295–0.2691)) than the subsocial *S. africanus* (0.0759 (95% CI: 0.0396–0.1210)) in the concatenated alignments of 12 496 codons in 23 immune genes (electronic supplementary material, table S4). A branch-site specific analysis of dN/dS ratios suggest that the elevated dN/dS averaged across sites is due to relaxed selection (RELAX: *k* = 0.62, *p* = 0.021, LR = 5.32). This is consistent with social immunity/protection leading to less infections of pathogens and/or a less efficient removal of slightly deleterious non-synonymous variants in the social *S. mimosarum*. This effect was not confirmed in dN/dS estimates of *S. dumicola* (0.1432 (95% CI: 0.0857–0.2285)) and *S. tentoriicola* (0.1827 (95% CI: 0.1096–0.2929)). In all four species, the differences in dN/dS estimates were mainly due to differences in rates of non-synonymous substitutions, since the rates of synonymous substitutions were similar in the two species pairs (electronic supplementary material, table S4 and S5). The estimates of the individual loci were highly variable, due to the existence of only few substitutions in many of the loci.

## 4. Discussion

Our results provide some evidence for less efficient immune responses of social species compared with subsocial sister species. This effect was strongest in the social *S. mimosarum,* and was coupled with elevated rates of non-synonymous substitutions in immune genes. We did not find evidence for increased selection on the immune response in social species, i.e. more rapid or stronger suppression of bacterial growth, as could be expected if social species experience elevated risk of social pathogen transmission. An impaired immune function in social species can be explained by two different hypotheses: an adaptive explanation suggests that social protection can lead to relaxed selection on immune responses. A non-adaptive explanation posits that elevated drift reduces the efficacy of selection to maintain an efficient immune system. Understanding the importance of an impaired immune function in social species requires separation of social protection and reduced efficacy of selection as the underlying force.

In social spiders, hygiene behaviour with removal of prey carcasses in social spiders (R Berger-Tal 2010, personal observation; [36]), and the presence of anti-microbial compounds from bacterial and fungal species in the communal nests of social *Stegodyphus* [7], which inhibit potential pathogens [37], could provide collective protection and potentially relax selection on the physiological immune response. Similar effects are seen in other social arthropods like termites and honeybees, where behavioural mechanisms may relax selective constraints on immune-related genes [12,38]. Common for these mechanisms are that the impaired immune function is adaptive and unlikely to reduce the persistence of social species. However, a 10-fold reduction in effective population sizes of social compared to subsocial spider species [22,39] substantiate the role of genetic drift in reducing efficacy of selection. Over time, this may reduce the ability of social species to combat pathogens through accumulation of slightly deleterious substitutions in immune-related genes. As expected under this scenario, previous studies found elevated rates of genome-wide non-synonymous substitutions in social species [29,30]. Here, our finding of elevated dN/dS ratios was likely caused by reduced efficacy of selection in one of the social species (*S. mimosarum*), while data from the other species pair were inconclusive. Note that if the inhibition of *B. subtilis* results from constitutively expressed immune effector molecules, the genes encoding signal transduction pathways components may not be involved in the immune responses tested here. Overall, we propose that reduced efficacy of selection is the most likely explanation for impaired immune function in social species.

Our finding that the transition to sociality may be accompanied by an impaired immune response may help explain frequent population and nest extinctions of social spiders [14,40–43]. Nest extinctions can be preceded by a build-up of cell bacterial load [41], supporting the hypothesis that a mounting parasite load contribute to high extinction rates of nests and entire populations after relatively few generations [19,41]. The ‘boom and bust’ population dynamics of social spiders further enhance the negative effects of low effective population size on loss of population and species-wide genetic diversity [21,22,28,39].

The superior inhibitory effect of *S. africanus* haemolymph relative to *S. mimosarum* on bacterial growth was immediate. Data on the *S. tentoriicola-S. dumicola* species pair were inconclusive, but possibly indicating a lower inhibitory effect on *B. subtilis* of the social *S. dumicola* after 20 min*.* This difference in timing suggests that the four species vary in the composition and/or concentration of inhibiting molecules in the haemolymph. Candidate molecules are the antimicrobial peptides (AMP) that contribute to bacterial inhibition by lysing cells within minutes of their release [44]. A broad suite of AMP genes are commonly upregulated in arthropods upon pathogen challenge, but may also be constitutively expressed especially in arachnids [25–27]. In spiders, they are stored in haemocyte granules in the haemolymph where they are released upon pathogen challenge [26,45,46]. Because AMPs are specific and potentially under diversifying selection (reviewed by [47,48]) they may contribute to between-species variation in inhibitory effects in *Stegodyphus*.

Evolutionary transitions from solitary to social living are often associated with decreased genetic diversity and efficacy of selection, and hence suggested to increase species' extinction risk [22]. Our results provide some support for reduced adaptive potential in immune responses of social *Stegodyphus.* They also highlight the potential role of pathogens as contributing and reinforcing drivers of meta-population dynamics by driving local extinctions, which further accelerates long-term loss of genetic diversity and thereby evolutionary potential.

## Data Availability

The data from this study are available from the Dryad Digital Repository: https://doi.org/10.5061/dryad.1ns1rn8x1 [49]. The data consist of the data from haemolymph assays, data for all spider species in assays with two different bacteria are uploaded in a single file (Step 2). We also present comparative analyses of sequence data of immune genes. All sequence alignments are uploaded as fas files (Step 2). The data are provided in electronic supplementary material [50].
